# Usefulness of Kidney Donor Profile Index (KDPI) to predict graft survival in a South Brazilian Cohort

**DOI:** 10.1590/2175-8239-JBN-2018-0263

**Published:** 2020-05-11

**Authors:** Natália Petter Prado, Cynthia Keitel da Silva, Gisele Meinerz, Roger Kist, Valter Duro Garcia, Elizete Keitel

**Affiliations:** 1 Santa Casa de Misericórdia de Porto Alegre, Serviço de Nefrologia e Transplante Renal, Porto Alegre, RS, Brasil.; 2 Universidade Federal de Ciências da Saúde de Porto Alegre, Programa de Pós-Graduação em Patologia, Porto Alegre, RS, Brasil.; 3 Universidade Federal de Ciências da Saúde de Porto Alegre, Programa de Pós- Graduação em Ciências da Saúde, Porto Alegre, RS, Brasil.; 4 Universidade Federal de Ciências da Saúde de Porto Alegre, Porto Alegre, RS, Brasil.

**Keywords:** Kidney Transplantation, Donor Selection, Graft Survival, Transplante de Rim, Seleção de Doadores, Sobrevivência de Enxerto

## Abstract

**Introduction:**

Kidney Donor Profile Index (KDPI) has been incorporated in the United States to improve the kidney transplant allocation system.

**Objectives:**

To evaluate deceased kidney donors’ profile using KDPI and compare to the previous United Network for Organ Sharing (UNOS) definition of expanded criteria donors (ECD) and assess the KDPI applicability to predict five-year graft survival and renal function in our sample.

**Methods:**

Retrospective cohort of 589 kidney transplants from deceased donors performed from January 2009 to May 2013 with follow-up until May 2018.

**Results:**

In 589 kidney transplants, 36.6% of donors were classified as ECD and 28.8% had KDPI ≥ 85%. Mean KDPI was 63.1 (95%CI: 60.8-65.3). There was an overlap of standard and ECD in KDPI between 60 and 95 and a significantly lower death-censored graft survival in KDPI ≥ 85% (78.6%); KDPI 0-20: 89.8%, KDPI 21-59: 91.6%, and KDPI 60-84: 83.0%; p = 0.006. The AUC-ROC was 0.577 (95%CI: 0.514-0.641; p = 0.027). Renal function at 5 years was significantly lower according to the incremental KDPI (p < 0.002). KDPI (HR 1.011; 95%CI 1.001-1.020; p = 0.008), donor-specific antibodies (HR 2.77; 95%CI 1.69-4.54; p < 0.001), acute rejection episode (HR 1.73; 95%CI 1.04-2.86; p = 0.034) were independent and significant risk factors for death-censored graft loss at 5 years.

**Conclusion:**

In our study, 36.6% were classified as ECD and 28.8% had KDPI ≥ 85%. KDPI score showed a moderate power to predict graft survival at 5 years. Renal function was significantly lower in patients with higher KDPI.

## Resumo

**Introdução:**

O Índice de Perfil de Doadores de Rins (KDPI) foi adotado nos Estados Unidos para melhorar o sistema de alocação de transplantes renais.

**Objetivos:**

avaliar o perfil dos doadores de rim falecidos usando o KDPI e comparar com a definição anterior do United Network for Organ Sharing (UNOS) de doadores de critérios expandidos (DCE) e avaliar a aplicabilidade do KDPI para prever a sobrevida do enxerto em cinco anos e a função renal em nossa amostra.

**Métodos:**

Coorte retrospectiva de 589 transplantes renais de doadores falecidos, realizada de janeiro de 2009 a maio de 2013, com acompanhamento até maio de 2018.

**Resultados:**

Em 589 transplantes renais, 36,6% dos doadores foram classificados como DCE e 28,8% apresentaram KDPI ≥ 85%. O KDPI médio foi de 63,1 (IC 95%: 60,8-65,3). Houve uma sobreposição de padrão e DCE no KDPI entre 60 e 95 e uma sobrevida do enxerto censurada por óbito significativamente menor no KDPI ≥ 85% (78,6%); KDPI 0-20: 89,8%, KDPI 21-59: 91,6% e KDPI 60-84: 83,0%; p = 0,006. A ASC-ROC foi de 0,577 (IC 95%: 0,514-0,641; p = 0,027). A função renal aos 5 anos foi significativamente menor de acordo com o aumento do KDPI (p <0,002). KDPI (HR 1.011; 95% CI 1.001-1.020; p = 0.008), anticorpos específicos contra doadores (HR 2,77; 95% CI 1,69-4,54; p <0,001), episódio de rejeição aguda (HR 1,73; 95% CI 1,04-2,86; p = 0,034) foram fatores de risco independentes e significativos para perda do enxerto censurada por óbito em 5 anos.

**Conclusão:**

Em nosso estudo, 36,6% foram classificados como DCE e 28,8% apresentaram KDPI ≥ 85%. O escore KDPI mostrou potencial moderado para prever a sobrevida do enxerto em 5 anos. A função renal foi significativamente menor nos pacientes com maior KDPI.

## Introduction

Expanded criteria donor (ECD) has been used in many medical centers, confronting the dilemma of accepting organs with expected lower allograft survival or discarding the organs and maintaining the patient on dialysis with a considerable mortality risk while waiting for another standard donor offer^1^^-^ 3. The 2001 United Network for Organ Sharing (UNOS) definition of ECD was developed as a binary variable to assess the risk of graft loss, however not all ECDs have the same risk. Trying to improve the prediction of ECD outcomes, an index was developed, namely the Kidney Donor Profile Index (KDPI), which is a numerical measure that combines 10 donor factors. This index is described as a percentile measure of which higher values are associated to worse outcomes^4^^,^ 5. Since 2014, KDPI has been used in the US kidney allocation system (KAS). The primary purpose of KDPI is the implementation of the “longevity matching” concept into the KAS. Candidates with longer estimated post-transplant longevity (EPTS score of 20% or less) will receive priority for kidneys from donors with a KDPI of 20%; on the other hand, donors with a KDPI ≥ 85% are thought to be equivalent to an ECD donor and are considered as a high-risk kidney^5^. In Brazil, an equation that quantifies the risk based on our donor profile does not exist. The aim of our study was to evaluate the profile of deceased kidney donors by using the KDPI calculator compared to the previous UNOS definition of ECD and the applicability to predict a five-year renal function and graft survival in our sample.

## Patients And Methods

This was a retrospective cohort study. The eligible population was all sequentially deceased donor kidney transplant recipients transplanted from January 2009 to May 2013 with a follow up until May 2018. Inclusion criteria were adult kidney recipients with at least 3 months of follow-up. We excluded kidney transplants (KTs) combined with another organ and all the cases with missing data necessary to calculate the KDPI scores. Clinical data from our local transplant database and medical records including baseline demographic characteristics from donors and recipients, transplant characteristics and clinical follow-up, return to dialysis, and death were collected. This study was undertaken following the principles stated by the World Medical Association Declaration of Helsinki and was submitted and approved by the Institutional Ethics Review Board.

Our kidney allocation system followed the human leukocyte antigen (HLA) compatibility score as a major weight variable. The crossmatch was performed for all transplants by cytotoxicity and flow cytometry. Our acceptance criteria of older donors followed the UNOS ECD definition associated with the pre-implant biopsy results. The biopsy criteria included the ECD donor, acute renal injury and macroscopy abnormalities.

For analyses purpose, the donors were classified by the previous UNOS criteria^6^ and KDPI using the formula available on the website of the OPTN (https://optn.transplant.hrsa.gov/resources/allocation-calculators/kdpi-calculator)^5^. KDPI was grouped based on its range for comparison, i.e., 0-20% for the 20% of the best donors and ≥ 85% for donors equivalent to ECDs in the US KAS. The intermediate range between 21-59% corresponded to the standard criteria donors (SCDs) while 60-84% range included the overlapped ECDs and SCDs.

Our sample did not involve any donor with cardiac death because Brazilian law does not allow it. The delayed graft function was defined as the necessity of at least one dialysis session within the first week after kidney transplantation. Donor specific antibodies (DSA) were considered for mean fluorescence intensity above 1000 in the pre-transplant sera screening in all recipients. Definition of acute rejection episode was biopsy-proved. The immunosuppressive regimen does not differ between SCD and ECD (anti-CD 25, tacrolimus, mycophenolic acid, and prednisone). Induction therapy with antithymocyte globulin was used in presence of DSA, panel reactive antibody above 50%, and cold ischemia time > 24 hours. The graft failure was defined as return to dialysis, preemptive re-transplantation, or death with functioning graft. The graft survival, death-censored graft survival, and glomerular filtration rate (GFR) after one, three, and five years post-transplantation were the evaluated outcomes. GFR was estimated by the chronic kidney disease epidemiology (CKD-EPI) formula^7^ and compared among the KDPI score ranges.

### Statistical analysis

Qualitative variables are presented as frequency and percentage. Quantitative variables with a normal distribution are reported as mean and standard deviation (SD) or 95% confidence interval (95%CI). The differences between mean scores were analyzed by the student *t*-test or ANOVA. Actuarial graft survivals were performed by the Kaplan-Meier and log-rank methods. The assessment of risk for graft loss was performed by the Cox uni and multivariate analyses and shown as hazard ratios (HRs) with 95%CI. In the multivariate analysis we included only the variables with a p value < 0.05 in the univariate analysis. We also performed the receiver operational characteristic (ROC) curves for assessing the predictive ability of KDPI to estimate the death-censored graft-failure. The value defined as cut-off was determined by the maximum of Youden index (J = sensitivity + specificity − 1). A p value < 0.05 was considered statistically significant. Statistical analysis was performed using SPSS V 23.0^8^.

## Results

During the study period, 744 deceased donor kidney transplants were performed of which 589 transplants fulfilled the inclusion criteria. Recipients aged less than 18 years (n = 126) and the kidney transplants combined with other solid organs (n = 29) were excluded. The demographic data are shown in Table 1.

**Table 1:** Demographic and clinical data.

Variable	N total = 589
Recipient Male gender	344 (58.4%)
Recipient age (years)	49.6 ± 12.5 (18–87)
Baseline disease	
Hypertension	98 (16.6%)
Diabetes Mellitus	88 (14.9%)
Polycystic Kidney disease	84 (14.3%)
Chronic glomerulonephritis	58 (9.9%)
Other	70 (11.9%)
Unknown (contracted kidneys)	191 (32.4%)
Transplant number	
First	527 (89.5%)
Two or more	62 (10.5%)
Positive HCV Receptor	57 (9.7%)
Panel Class I	
Zero	256 (43.4%)
1 a 10	148 (25.1%)
11 a 50	115 (19.5%)
51 a 80	35 (6%)
Upper to 80	35 (6%)
Panel Class II	
Zero	275 (46.7%)
1 a 10	150 (25.5%)
11 a 50	113 (19.2%)
51 a 80	29 (4.9%)
Upper to 80	22 (3.7%)
Mismatch (n = 564)	
Zero	17 (3%)
1 a 3	382 (67.7%)
4 a 6	165 (29.3%)
Presence of DSA	98 (16.6%)
Positive Flow B Cross Match (n = 463)	21 (4.5%)
Acute Rejection (n = 588)	79 (13.4%)
Cold Ischemia Time (hours)	23.75 ± 6.67
Delayed graft function (n = 419)	273 (65%)
Dual kidney implantation	21 (3.6%)
Donor Age (year)	46.2 ± 17.2 (1–79)
Male donor gender	358 (60.8%)
Positive HCV Donor	20 (3.4%)
ECD UNOS criteria (binary)	214 (36.3%)
KDPI	
Mean (95% CI)	63.1 (60.8–65.3)
Median	69
Local Donor (n = 460)	325 (55.2%)
Induction	
No induction	114 (19.4%)
IL-2Ri (Basiliximab)	314 (53.3%)
ATG (Thymoglobulin)	158 (26.9%)
ATG + Plasmapheresis + IV Immunoglobulin	2 (0.3%)
Plasmapheresis + Rituximab	1 (0.1%)
Maintenance Immunosuppression protocol	
FK + Mycophenolate + Prednisone	552 (93.7%)
CyA + Mycophenolate + Prednisone	24 (4.1%)
Others	13 (2.2%)

HR: hazard ratio; CI: confidence interval; KDPI: Kidney Donor Profile Index; CIT: cold ischemia time; DSA: donor specific antibody; BPAR: biopsy proven acute rejection; PRA panel reactive antibodies; ECD UNOS: Expanded Criteria Donor by United Network for Organ Sharing; Rec Age: recipient age; DGF: delayed graft function.

* Outcome/At risk.

**Figure 1:**
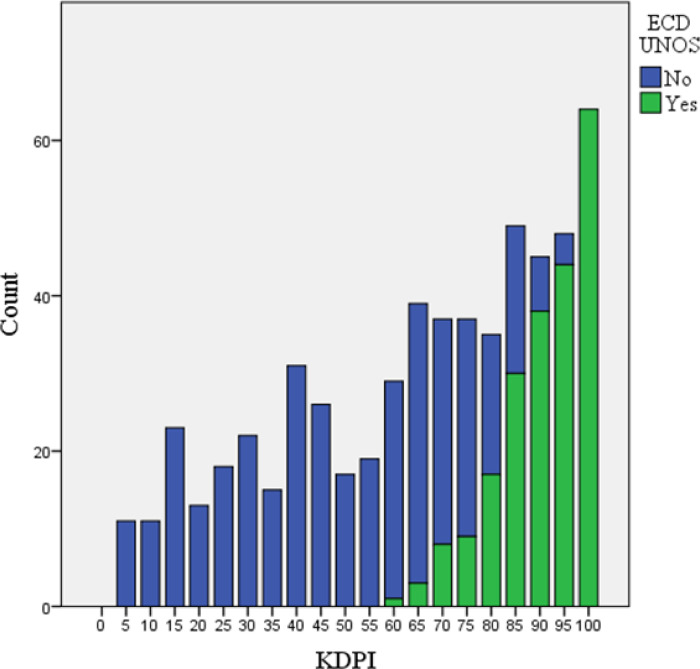
Donors’ count according to KDPI and ECD (UNOS) criteria. Note: KDPI: Kidney Donor Profile Index; ECD UNOS: Expanded Criteria Donor defined by United Network for Organ Sharing.

Kidney transplants from ECDs by the previous UNOS criteria were 36.3%, and 28.8% had KDPI ≥ 85%. The mean KDPI was 63.1 (95%CI: 60.8-65.3). When comparing donors according to the KDPI and UNOS criteria, all KDPIs inferior to 60 were considered as standard criteria donor (SCDs) and all KDPI that equaled or exceeded 95% were regarded as ECDs. There was an overlap of SCDs and ECDs in KDPI between 60 and 95% (Figure 1).

The mean recipient age (years) in the KDPI categories was 0-20: 44.79 (95%CI: 41.1-48.4); 21-59: 45.45 (95%CI: 43.6-47.2); 60-84: 49.96 (95%CI: 48.2-51.7) and ≥ 85: 55.2 (95%CI: 53.6-56.8). There was a significant difference in recipient age between KDPI ≥ 60 and ≥ 85 when compared to KDPI < 60 (p = 0.002 and p < 0.001, respectively). There was no significant difference when comparing recipient age with KDPI between 0-20 and 21-59%.

**Figure 2:**
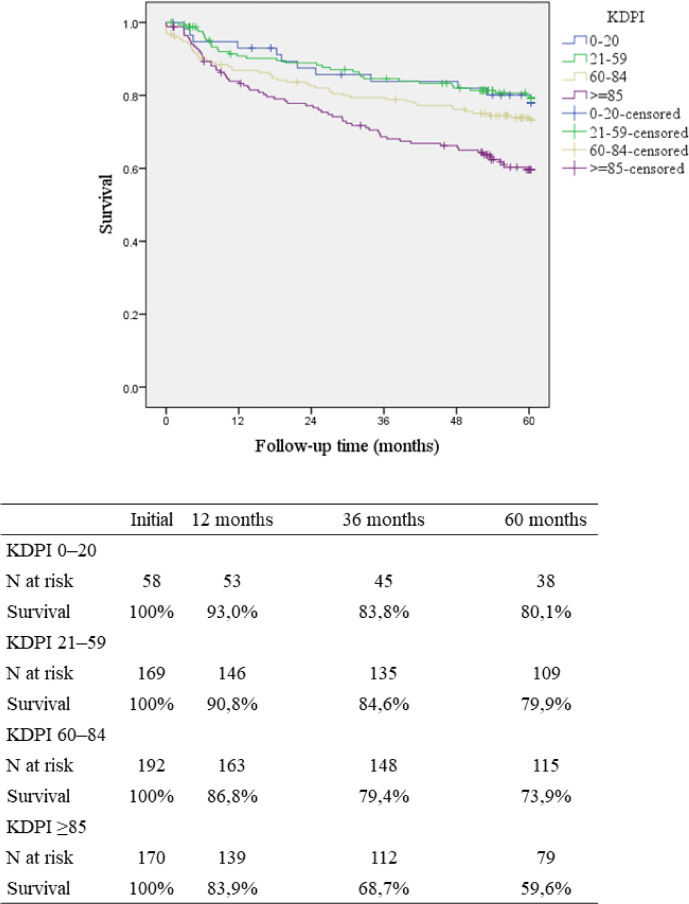
Graft survival according to KDPI ranges. Note: KDPI: Kidney Donor Profile Index.

The global graft survival according to KDPI is presented in Figure 2. There was a significantly lower 5-year graft survival for KDPI ≥ 85% (59.6%) when compared to the other ranges (KDPI 0-20: 80.1%; KDPI 21-59: 79.9% and KDPI 60-84: 73.9%; p < 0.001). There were 82 deaths in the study period. The main causes were infection (n = 49), cardiovascular (n = 13), and neoplasm (n = 7). The causes of return to dialysis (n = 70) were immunological (n = 36), infection (n = 18), surgical (n = 3), recurrence of original disease (n = 2), and others (n = 11). Nine grafts had primary nonfunctioning.

**Figure 3:**
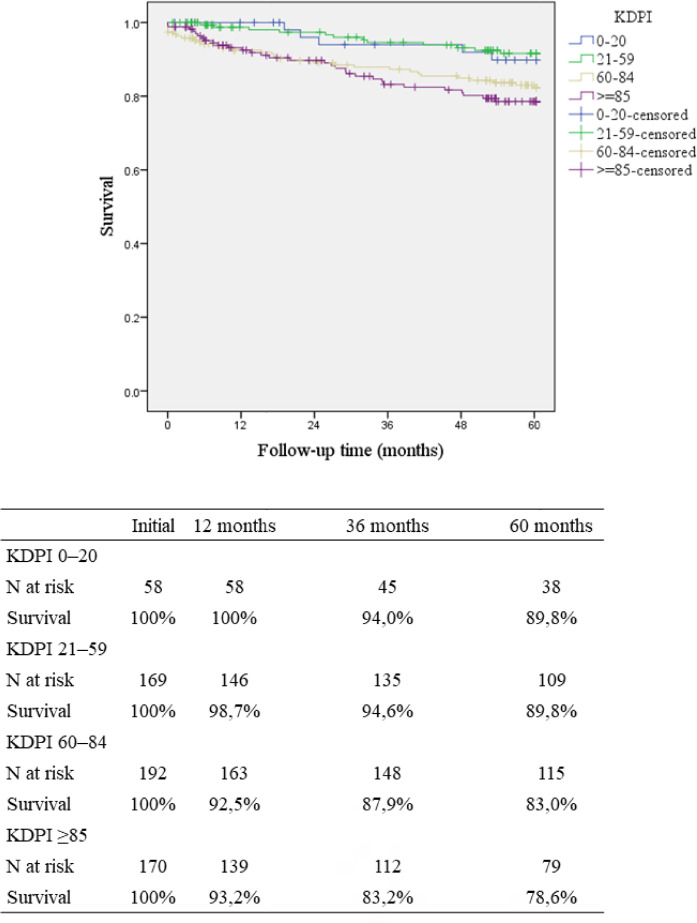
Death-censored graft survival according to KDPI ranges. Note: KDPI: Kidney Donor Profile Index.

There was also a significantly lower 5-year death-censored graft survival in KDPI ≥ 85 (78.6%); KDPI 0-20: 89.8%, KDPI 21-59: 91.6%, and KDPI 60-84: 83.0%; p = 0.006 (Figure 3).

The AUC-ROC for graft loss was 0.577 (95%CI: 0.514-0.641; p = 0.027). KDPI of 71% presented the best sensitivity (55.7%) and specificity (55.7%). The KDPI of 85% showed 39% sensibility and 73% specificity for 5-year death-censored graft loss.

**Figure 4:**
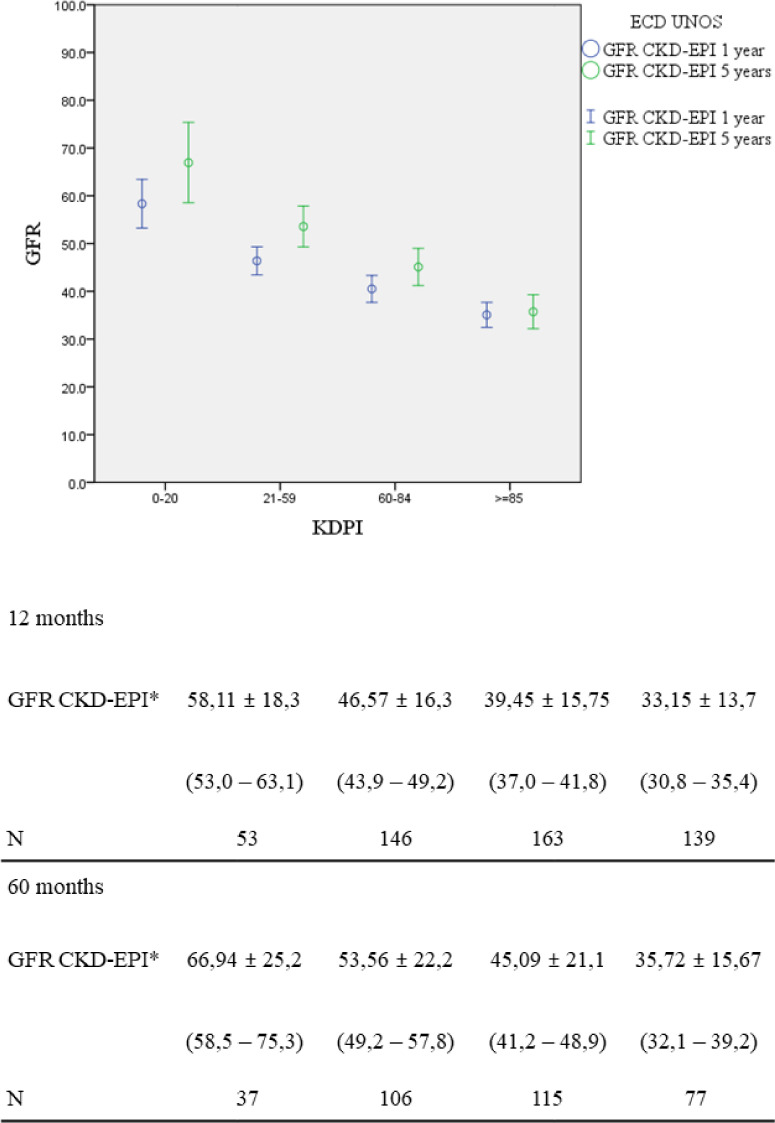
Renal function at 1 and 5 years post-transplantation according to KDPI ranges. Note: GFR: Glomerular Filtration Rate (mL/min/1.73m2); KDPI: Kidney Donor Profile Index; CKD-EPI: Chronic Kidney Disease-Epidemiology Collaboration. *p<0.02 for all groups compared among them.

Kidney function at 1 and 5 years of follow-up was significantly lower with higher KDPI (p < 0.002) (Figure 4).

The univariate analysis for the risk of death-censored graft loss is presented in Table 2. For each KDPI incremental point there was a 1.1% (HR 1.01; 95%CI 1.003-1.020 p = 0.011) increased risk of 5-year graft loss. Kidneys from ECDs defined by the UNOS criteria presented 207% increased risk for graft loss when compared to SCDs (p = 0.001). The presence of DSA also showed an increased risk (HR 2.48; 95%CI 1.54-3.99; p < 0,001). Among 98 recipients with DSA, 36 (36.7%) died or returned to dialysis in 5 years after transplantation when compared to 123 (25.3%) among 487 without DSA. The pre-transplantation T cell flow cytometry crossmatch was negative in all recipients; however, 21 had positive B cell flow crossmatch. The later was a risk factor for graft loss (HR 2.64; 95%CI 1.13-6.17; p = 0.002). The PRA class II above 80% increased the risk of graft loss (HR 3.24; 95%CI 1.41-7.41; p = 0.005). For each additional hour of cold ischemia time, the risk of graft loss was increased by 6% (CIT measured in minutes: HR 1.001; 95%CI 1.00-1.001; p = 0.032.). The recipient age and delayed graft function were not significant risk factors for graft loss.

**Table 2:** Univariate and multivariate analysis for 5-year death-censored graft loss risk.

	N*	Univariate			Multivariate		
		HR	CI (95%)	p	HR	CI (95%)	p
KDPI	79/589	1.01	1.00–1.02	0.011	1.011	1.003–1.020	0.008
CIT (min)	76/564	1.001	1.000–1.001	0.032	1.000	1.000–1.001	0.161
DSA	78/585	2.48	1.54–4.00	0.000	2.77	1.69–4.54	< 0.001
BPAR	79/588	1.85	1.14–3.01	0.013	1.73	1.04–2.86	0.034
PRA II (%)	78/586						
Zero	272						
1–10	150	1.37	0.79–2.40	0.264	1.24	0.69–2.22	0.478
11–50	113	1.67	0.93–2.98	0.086	1.42	0.76–2.67	0.274
51–80	29	0.61	0.14–2.56	0.498	0.40	0.09–1.80	0.236
> 80	22	3.24	1.42–7.42	0.005	1.75	0.65–4.71	0.265
ECD UNOS	79/589	2.07	1.33–3.22	0.001			
Rec Age	589	1.00	0.98–1.02	0.964			
DGF	419	0.88	0.50–1.55	0.662			
PRA I (%)	586						
Zero	272			0.070			
1–10	150	0.62	0.32–1.20	0.154			
11–50	113	1.37	0.79–2.39	0.262			
51–80	29	0.83	0.29–2.35	0.730			
> 80	22	1.98	0.95–4.14	0.069			

HR: hazard ratio; CI: confidence interval; KDPI: Kidney Donor Profile Index; CIT: cold ischemia time; DSA: donor specific antibody; BPAR: biopsy proven acute rejection; PRA panel reactive antibodies; ECD UNOS: Expanded Criteria Donor by United Network for Organ Sharing; Rec Age: recipient age; DGF: delayed graft function.

* Outcome/At risk.

The multivariate analysis is also shown in Table 2. The PRA Class II and CIT lost significance (p = 0.350 and 0.161, respectively). KDPI (HR 1.00; 95%CI 1.00-1.02; p = 0.008), DSA (HR 2.77; 95%CI 1.69-4.54; p < 0.001), and acute rejection episode (HR 1.73; 95%CI 1.04-2.86; p = 0.034) remained as an independent and significant risk factors for death-censored graft loss at 5 years.

The previous UNOS criteria were not included in the multivariate analysis because they are strongly correlated with KDPI; thus, the 4 variables in the UNOS criteria for EDC were included in KDPI.

## Discussion

In our cohort, 36.3% of kidney transplants were from ECDs matching the UNOS criteria and 28.8% had KDPI ≥85%. In comparison, an US study found 17.3% ECD and 9.7% KDPI > 85%^9^ while a Spanish study showed 41.9% ECD and 35% KDPI ≥ 85%^10^. A South Brazilian study of 346 renal transplants found a 30.6% ECD^11^. Studies from other Brazilian regions presented 9.4% (Ceará) and 16.5% (São Paulo) of transplants using ECD^12^^,^ 13. In our sample, 45% of renal transplants were allocated from other states of Brazil. This means a higher ECD acceptability of our state when compared to other states in Brazil, which is similar to the Spanish model^10^^,^ 12 ^,^ 13.

Our study demonstrated a considerable overlap in the KDPI distribution between SCD and ECD categories also noticed by Rao et al. and Woodside et al. This overlap was in KDPI range between 60 and 95%^4^^,^ 14. The KDPI represents a substantial improvement in scale and interpretability relative to the less accurate SCD versus ECD classification. It was developed to improve the organ allocation and decrease kidney discard. Our sample allografts from ECD had HR 2.07 5-year death-censored graft loss when compared to SCD. Molnar et al. and Reeves-Daniel showed HR of 1.82 and 1.45, respectively^15^^,^ 16. American studies found the relative risk of 1.7 to 1.77 for graft loss in ECD transplants^17^^-^ 19. Mezrich et al. showed HR equal to 1.49 (95%CI 0.98-2.27) of graft failure and patient death for ECD kidney recipients (not significant for recipients between 40 and 59 years)^20^. The justification for the use of ECD is the beneficial effect when compared to the patients remaining on the waiting list, especially those older than 40 years and with diabetes, who would most likely not survive long waiting periods^3^^,^ 21 ^-^ 23.

In our study, each KDPI increment of 1% was independently associated to 1% graft loss risk at 5 years in univariate as well as multivariate analyses. Arias-Cabrales et al. found 3% increase in the graft loss risk for each KDPI increment^10^. Gandolfini et al. observed higher KDPI values associated to poorer graft outcomes in an Italian cohort of 442 marginal kidneys allocated as single or dual kidney transplantation^24^. Woodside et al. found a similar graft survival for ECD and SCD in each KDRI range^14^. Other authors also showed a relationship between higher KDPI, risk of graft failure, and recipient death^25^^-^ 27. The KDRI has already been validated in the Dutch population^28^. In Spain, the KDPI and KDRI were validated for ECDs^29^.

Despite the acceptable graft survival results, the discard rates have increased in US, especially for high KDPI kidneys. From 2012 to 2014, 18.3% of available kidneys for transplant were discarded. The discard rates increased 50.6% for KDPIs > 80% and 71.6% for KDPI > 95%^30^. In Europe, the discard rate was 14% in the same period^31^. This difference may be partially due to the “fear of flagging” worse graft and patient survival in the program-specific reports on kidney transplantation in US; these underperforming programs may have a penalty^27^. In 2017, in Brazil, we estimated an overall kidney discard rate of 30% and there is no register for donor risk index^32^.

Lehner et al. observed a GFR reduction as KDPI increased (65.8, 60.4, 46.1, and 35.2 mL/min/1.73 m^2^ for KDPI < 20, 21-34, 35-85, and > 85%, respectively). The reduction rate was similar in the different groups during the follow-up; however, they included a GFR of 0 mL/min/1.73 m^2^ for patients with graft loss^26^. In our study we also observed a lower GFR in different KDPI groups, but we included in the analysis only functioning grafts. The GFR decrease at 5 years was not observed, probably due to the fact that the patients with worse graft function lost the graft before 5 years, therefore less patients had GFR measured at 5 years.

The other independent risk factors for graft loss, namely DSA and acute rejection episodes, were defined in other studies^33^^-^ 37.

The mean cold ischemia time was around 24 h, similar to other studies in Brazil^38^^,^ 39, but superior to the US (14.2 to 17.9 h)^40^ and Europe (18 h)^41^^,^ 42 reports. In our sample, 21.3% of transplants had CIT superior to 24 h, which may contribute to our high rate of DGF (63.5%). This DGF rate is similar to the ones obtained in the other Brazilian studies, ranging from 54.2% to 70.8%^11^^-^ 13 ^,^ 43, but much higher than presented in international studies^33^^,^ 40 ^,^ 44 ^,^ 45. The CIT and DGF were not independent risk factors for graft loss but they could have contributed for the very high rate of acute rejection in our sample.

The strength of our study was to include a large number of kidney recipients while a retrospective single-center report was its limitation.

The predictive power of KDPI in our sample was moderated with an AUC-ROC of 0.577 (0.514-0.641; p = 0.027), which is similar to the results in the literature (C Statistic = 0.60 in the US). Thus, it is not precise enough to estimate with high confidence the quality of donors with close KDPI scores. Furthermore, the KDPI does not include other donor risk factors related to outcomes as pre-implant biopsies and kidney anatomical alterations (damage, atherosclerotic lesions, and cysts). The KDPI also does not include the probability of cancer and infection transmissions, except hepatitis C. In our sample there was no donor with cardiac death because in our country it is not allowed.

## Conclusion

In our study, 36.6% of kidney donors were classified as ECDs and 28.8% had KDPI ≥ 85%. There was an overlap of ECD/SCD with KDPI scores between 60 and 95%. All KDPI scores above 95% were considered as ECDs.

The KDPI score showed a moderate accuracy to predict graft survival at 5 years. For each KDPI increment point there was an increased graft loss risk of 1%. Recipient immunological variables were more accurate to predict graft survival.

The GFR was significantly lower for higher KDPI scores at one and five years after transplantation. The KDPI calculator is available for free online and does not cause any delay in kidney allocation. It seems to be a useful tool to help in organ allocation, even though it was developed based only on the North American population.

Ideally, there should be an equation based on Brazilian donors to apply in our population.
